# Scale-up of prevention programmes: sustained state-wide use of programme delivery software is explained by normalised self-organised adoption and non-adoption

**DOI:** 10.1186/s13012-021-01184-2

**Published:** 2022-01-15

**Authors:** Eileen Goldberg, Kathleen Conte, Victoria Loblay, Sisse Groen, Lina Persson, Christine Innes-Hughes, Jo Mitchell, Andrew Milat, Mandy Williams, Amanda Green, Penelope Hawe

**Affiliations:** 1 Sax Institute, Sydney, Australia; 2 Menzies Centre for Health Policy and Economics, University of Sydney, D17, Sydney, NSW 2006 Australia; 3 University of Southern Denmark, Esbjerg, Denmark; 4 New South Wales Ministry of Health, Sydney, Australia; 5 The Australian Prevention Partnership Centre, Sax Institute, Sydney, Australia; 6 South Western Sydney Local Health District, Sydney, Australia

**Keywords:** Programme scale-up, Self-organisation, Complexity, Normalisation, Practice values, Practice system

## Abstract

**Background:**

Population-level health promotion is often conceived as a tension between “top-down” and “bottom-up” strategy and action. We report behind-the-scenes insights from Australia’s largest ever investment in the “top-down” approach, the $45m state-wide scale-up of two childhood obesity programmes. We used Normalisation Process Theory (NPT) as a template to interpret the organisational embedding of the purpose-built software designed to facilitate the initiative. The use of the technology was mandatory for evaluation, i.e. for reporting the proportion of schools and childcare centres which complied with recommended health practices (the implementation targets). Additionally, the software was recommended as a device to guide the implementation process. We set out to study its use in practice.

**Methods:**

Short-term, high-intensity ethnography with all 14 programme delivery teams across New South Wales was conducted, cross-sectionally, 4 years after scale-up began. The four key mechanisms of NPT (coherence/sensemaking, cognitive participation/engagement, collective action and reflexive monitoring) were used to describe the ways the technology had normalised (embedded).

**Results:**

Some teams and practitioners embraced how the software offered a way of working systematically with sites to encourage uptake of recommended practices, while others rejected it as a form of “mechanisation”. Conscious choices had to be made at an individual and team level about the practice style offered by the technology—thus prompting personal sensemaking, re-organisation of work, awareness of choices by others and reflexivity about professional values. Local organisational arrangements allowed technology users to enter data and assist the work of non-users—collective action that legitimised opposite behaviours. Thus, the technology and the programme delivery style it represented were normalised by pathways of adoption *and* non-adoption. Normalised use and non-use were accepted and different choices made by local programme managers were respected. State-wide, implementation targets are being reported as met.

**Conclusion:**

We observed a form of self-organisation where individual practitioners and teams are finding their own place in a new system, consistent with complexity-based understandings of fostering scale-up in health care. Self-organisation could be facilitated with further cross-team interaction to continuously renew and revise sensemaking processes and support diverse adoption choices across different contexts.

Contributions to the literature

- It is rare to apply the Normalisation Process Theory to describe implementation on a population scale outside clinical settings.
- Researchers usually describe how a recommended practice embeds or normalises, not how non-compliance also normalises, as we have done here.
- Self-organisation (from complexity theory) accounts for how teams support each other’s decisions not to use a technology, enabling state-wide implementation targets to be reportedly reached, nonetheless. This is a “first” in terms of observation.
- While seemingly contrary, when preferred practice styles and professional values are diverse, high tolerance of individual non-use of a recommended technology might underpin its success.

## Introduction

Over the last two decades, there has been an increasing effort to implement efficacious interventions “at-scale” [1, 2]. Electronic implementation monitoring systems have evolved to track their delivery. A large body of literature evaluates the implementation and integration of electronic health systems in various clinical contexts [3]. However, there is currently little research examining how e-monitoring systems have enabled population-level prevention, and why some systems do not endure while others do better [4].

The Normalisation Process Theory (NPT) is a sociological theory developed to explain why something novel becomes regular or routine [5–7]. NPT focusses on why interventions have either been successfully embedded and sustained, have altered course, or instead have failed to replace existing practice. NPT has been used extensively in clinical contexts [8–11]. In a systematic review, McEvoy and colleagues [12] noted that NPT may enhance the capacity to design interventions and explore mediating pathways that shape and improve implementation processes. More recently, Carl May (the NPT founder) and colleagues have observed the use of NPT across a range of different types of in-hospital, primary care and community interventions [13]. In all but one study, the outcomes of implementation could be explained by reference to the four main mechanisms specified within NPT. These main mechanisms are coherence (how practitioners make sense of the innovation, how it comes to be specified, differentiated and internalised as a practice); cognitive participation (how practitioners initiate and enrol in the behaviour, how it becomes legitimised); collective action (how teams make the new practice work, how work is organised and structured); and reflexive monitoring (how the new practice is appraised/ evaluated by practitioners and how this informs the reconfiguration of new practice).

Our research objective was to use NPT as a template for appreciating the uptake and embedding of technology-assisted obesity prevention programme delivery into schools and childcare settings in New South Wales (NSW). It is Australia’s largest single investment in childhood obesity prevention. We were guided by others’ use of NPT in related contexts, that is, settings where a purpose-built technology or software system was assisting the implementation of health-related programmes [9, 10, 13–15].. We draw on ethnographic descriptions and reflections from programme implementers to understand how the activities required by the software embedded. We focus on the integration into day-to-day work, rather than the achievement of programme goals. Our aim is to understand how new scale-up technology was being used across the state and its fit within local organisational practice.

## Context

The Healthy Children Initiative (HCI) includes the scale-up of two evidence-based childhood obesity prevention programmes in NSW with the aim of improving healthy eating and physical activity [16, 17]. By mid-2019, 88% of all child care centres and 83% of all primary schools were reported to be taking part [unpublished data]. This represents the largest ever prevention programme scale-up in Australia’s history.

The Population Health Information Management System (PHIMS) is an electronic monitoring system, purpose-built to support the implementation of HCI. PHIMS provides a platform for state-wide monitoring (i.e. evaluation) and reporting against agreed targets for implementation i.e. the proportion of sites (schools and childcare centres) that have made the recommended policy and practice changes. The use for this purpose is mandatory. Additionally, at the local level, it is also recommended that health promotion practitioners use PHIMS to track and plan visits to sites to document their interactions with sites and to record site-level progress towards achievement of a set of specified healthy eating and physical activity practices (e.g. site has a nutrition policy). Data about practice achievement per site is available for local health districts, while the Ministry of Health has access to data in aggregated form for state-wide evaluation purposes. Global funding service agreements require local health districts to meet targets regarding the amount of organisational practice change achieved in their sites.

The system’s longevity is unusual as elsewhere purpose-built software technology to assist large-scale prevention programme scale-up has not been sustained [4]. This provided the impetus for our case study. What are the dynamics associated with PHIMS use? The study we report on here is a part of a larger investigation of this question [18] within a university-policy-maker-practitioner partnership centre [19]. Earlier papers report on the data recording methods that have grown up alongside PHIMS [20]; the style of practice we observed [21]; the breadth and intensity of work to achieve a change in nutrition or physical activity policy/practice in a site [22]; the role of programme materials [23]; the co-production methods within our team [24, 25]; and reactions from PHIMS designers and administrators to a preliminary “rich picture” of how PHIMS might work as a system [26].

## Methods

This cross-sectional study forms part of a multi-site ethnography undertaken within all health promotion teams in NSW. The research was described to psotential participants as an opportunity to learn about how the HCI has been implemented and how PHIMS is being used. A detailed description of the protocol has been published elsewhere [18].

Three ethnographers (KC, VL, SG) conducted short-term, high-intensity ethnographic observation of programme implementation teams consistent with focussed ethnography methods [27]. Our fieldwork was conducted from August 2017–Aug 2018 when PHIMS had been in place for 4 years. The ethnographers accompanied practitioners as they went about their day-to-day health promotion practice; witnessing the interactions between practitioners and sites. They sat in the offices while practitioners scheduled appointments and fielded phone calls, sat adjacent while data was entered into PHIMS and attended meetings and training sessions on PHIMS. They engaged in conversational interviews [28] and compiled extensive field notes, materials and audio-recordings [29]. The representation of findings from fieldwork was iteratively critiqued and interpreted within the research team, with participants and others involved in the design and planning of PHIMS.

The team used a grounded theory approach [30] and NVivo qualitative data analysis software [31] to generate an initial codebook and to sort data. For this paper, we extracted all the “PHIMS” subcodes from across all teams (see Appendix 1). One researcher (EG), supervised by (PH), applied a template analysis [32], reading through all of the material in the PHIMS codes and categorising the data into the four a priori themes of NPT (i.e. coherence/sensemaking; cognitive participation; collective action; and reflexive monitoring) as outlined in Appendix 2, informed by template analyses using NPT (e.g. [33]). EG, KC and PH collectively coded and shared interpretations of an initial sample of data. Following refinement and practice, NPT template coding was conducted by EG (conferring with PH). Note that we used the NPT constructs to describe *how* PHIMS might have become embedded. We did not use NPT, to devise scores on the NPT dimensions (e.g. [34]).

After the initial findings were discussed, additional analyses were undertaken to confirm (or contradict) initial findings (triangulation) [18]. The finding that practitioners appraise PHIMS according to how it fits with their practice values and what they feel health promotion “ought” to be required substantial exploration. This finding had significant overlap with the initial codebook developed through the grounded theory approach, where extensive data was coded under “Values and attitudes to practice”. VL extracted and read all the data under this code, identifying and sorting the corroborating/or disconfirming evidence. The evidence from this additional analysis was read in entirety by a second researcher PH and the insights added to the overall findings. All names are pseudonyms. We used the consolidated criteria for reporting qualitative research (COREQ) guidelines [35] to guide the reporting of our study (see Additional file 1).

## Findings

All 14 HCI teams took part. We present our results using the four main themes of NPT as a guide. Those practitioners who had been around since the inception of PHIMS easily recounted stories of what it was like when it was first introduced. We use quotation marks to depict verbatim statements from participants; fieldnotes are written from the point of view of the ethnographer.

Like others, we found that NPT coding categories occasionally overlapped, but this did not temper our ability to explore and interrogate the meaning of a field note or quotation [13]. NPT allowed us to consider the way people thought about and enacted PHIMS both individually and collectively. We found that while some teams conveyed a fairly uniform way of thinking about and enacting PHIMS, in other places different practitioners in the same team had different attitudes and practices regarding PHIMS. We observed embedding taking place (or not) in multiple ways. In some cases, PHIMS was regarded as an asset and an accepted way of doing things. In other cases, PHIMS use appeared to be incorporated in a minimal way only. Even where PHIMS was less well regarded or apparently underutilised, we found people reported practice activities had altered. Table 1 summarises our findings overall.

**Table 1:** Summary of results

NPT core mechanism	Further description	Findings
Coherence/sensemaking	How PHIMS is understood; how it is differentiated from previous ways to do the same thing; how the work is specified; how the meaning is internalised.	• PHIMS appears to authorise and legitimise practice because it scales up two evidence-based programmes • Practitioners give clear “before-and-after” accounts, i.e PHIMS was a large-scale transformative event • There is a spectrum of PHIMS use. At one end some are using it only to comply with mandatory reporting of the extent to which sites are achieving the designated practices (e.g. food policy at school). While at the other end PHIMS is used to structure and direct how they do their practice (e.g. following PHIMS prompts to encourage food policy at schools)
Cognitive participation	How practitioners become engaged and take part in PHIMS work. How PHIMS work is legitimated; how practitioners are recruited/enrolled as PHIMS users and how involvement is sustained over time.	• Technical or training barriers can be associated with a lack of engagement in PHIMS. • Practitioner experience of PHIMS may be influenced by higher level (HCI and local district) management style and attitudes. • PHIMS champions incorporate a personalised one-to-one approach in training others • Missed opportunities for legitimation: some practitioners say they would be more engaged in PHIMS if they could see and understand how the central administrators benefited from their data.
Collective action	How people come together (interact) to make the PHIMs work; organisation, structure and workability	• Time taken and technical skills in using PHIMS have become part of health promotion practice • New roles related to PHIMS use have been created and maintained within teams (e.g. data entry). • Defining minimum standard for what should be entered into PHIMS also acts to define practice. • In some sites, it’s one person’s job to enter data into PHIMS on behalf of others (the non-users). • Use of PHIMS data for team-level planning has changed the course of practice in some teams. • The colloquial language of “PHIMS guru” illustrates a form of everyday informal integration.
Reflexive monitoring	How PHIMS is appraised; opportunities to view its benefits/costs	• The percentage of sites reaching recommended practice targets can be calculated for each team • Many practitioners appreciate the significance of health promotion across the state being made more visible through PHIMS. More could be made of this. • PHIMS makes it possible to gain instant feedback about how some forms of progress are made, allowing tailoring, coordination and adjustment of work • Criticism of what PHIMS represents (“mechanisation” or over-standardisation of health promotion) serves to articulate practice values i.e. what health promotion should be (and what PHIMS is not). Some practitioners devise ways to do what is required for reporting in PHIMS without compromising what they feel is the best way to work

### Coherence: making sense of PHIMS

In NPT, *coherence* refers to the sense individuals make of a new technology or innovation in relation to the usual work or practices that have occurred prior. We used this construct to look for evidence that there was a distinct boundary when PHIMS started and how that was interpreted.

Practitioners compared and differentiated PHIMS to the systems used prior to its introduction.

“I just couldn’t believe it when I came on board, everyone had their own spreadsheets. It was a bit of a mess to be honest.” Everyone was recording different things. At least (now) with PHIMS it’s more systematic and they’re all recording the same kind of data. (Fieldnotes, LHD D)

In some teams, practitioners described how PHIMS had changed the way they work. Some explained that it allowed them to build and strategically direct their practice, providing “an evidence base of what we’re doing.” Others used PHIMS to track their progress in the delivery of programmes, helping them decide where to focus their work. One team used PHIMS to track staff performance and see where staff were not keeping records up to date.

Some practitioners valued the ability to enter detailed qualitative information in PHIMS as a way of tracking their contact and interactions with services over time, building fluid relationships and continuity of workflow among different team members. This was considered an aspect of coherence because the new technology aligned with pre-existing ideas of the best ways to manage work and teams. However, some practitioners kept separate notes on their practice and viewed PHIMS as primarily a tool for external accountability purposes such as recording the achievement of a practice change (an implementation target).

PHIMS was designed to be used by health promotion practitioners to record the extent to which a school and childcare centre is implementing HCI programmes. It was intended to be used at a district level by managers who oversee the implementation of programmes across a whole district, and at a state level by policy-makers, and administrators who use PHIMS to assess the implementation of programmes across the whole state. However, the utility of these functions was dependent on how much information individual practitioners entered into the system.

Numerous practitioners said that there was a lack of formal communication around the role and purpose of PHIMS. This seemed to affect their willingness to integrate the technology into their practice:

Ava says that it would help if the Ministry communicated... more... If they knew that the Ministry was able to get their contracts renewed for example because of PHIMS data, they would probably feel much better about it. She relates this to Abigail: “Don’t you think you would use PHIMS better if you knew that the Ministry was able to get more money for HCI because of it?” Abigail agrees. (Fieldnotes, LHD D)

Some practitioners conveyed uncertainty about the value of reporting in PHIMS:

(Tilly) acknowledges that she wasn’t properly trained in PHIMS, and now is learning that there may be some things that could help her, or that she is supposed to be reporting … Tilly doesn’t seem particularly against using PHIMS or entering data into PHIMS, she just seems to be befuddled about how the data is used and who it is benefitting. (Fieldnotes, LHD N)

Tilly seemed unconvinced that using PHIMS was something she needed to do better at her job and continued using her own system to manage her practice. In this way, a lack of clarity about PHIMS’ purpose could affect the extent to which a practitioner used PHIMS.

### Cognitive participation: commitment to and engagement in the use of PHIMS

A focus on the second construct in NPT, *cognitive participation*, allowed us to consider how practitioners commit to using PHIMS both individually and collectively. We found some legitimised PHIMS’ role in their practice. Others legitimised a marginal role for PHIMS.

Early negative experiences threatened PHIMS use, demonstrating cognitive non-participation. It was common to talk about technical difficulties and perceived flaws in PHIMS functionality and the lack of an updated intuitive user interface. Practitioners would sometimes describe it as “clunky” or “structured and tedious.” One team spent a long time collectively working past the “kinks” in PHIMS:

They say they’ve had a lot of frustration with PHIMS, they didn’t always like it and they don’t always like it but PHIMS has improved a lot over time and they have figured out how to work in it. (Fieldnotes, LHD I)

In another team, a practitioner who had integrated PHIMS effectively into their work practices advocated for PHIMS, persuading other team members of the benefits of seeing progress at the state level and knowing the impact of their work.

We found that the way practitioners engaged with PHIMS was influenced to an extent by the HCI management style present in different sites. Certain managers required staff to follow the PHIMS schedule of activities precisely and focused on KPIs. Practitioners in these teams were likely to have a different experience with PHIMS than if staff were told to use PHIMS as a tool for reporting purposes only. One HCI manager worked to “unhitch” PHIMS from practice. Yet, within that team, one practitioner described PHIMS as “useful”:

"I feel like it’s a patient chart … you can open a site and you can get everything you want about it. I personally use PHIMS in that way.” (Interview, LHD F)

In some teams, practitioners were grappling with inconsistencies in terms of the quantity and quality of qualitative information that individuals input into PHIMS. With the onus on individuals to commit to using the technology and to decide how they will use it, some users put detailed information in the system while others barely used it. This posed a problem in cases where the responsibility for working with particular schools shifted between team members who then found themselves reliant on PHIMS data in order to understand the ongoing relationship and progress of work with schools.

### Collective action: how practitioners integrate PHIMS in practice

The third construct in NPT, *collective action*, directed our attention to the ways health promotion practitioners interact and organise themselves to integrate PHIMS into their everyday work. In some contexts, individuals or groups of practitioners made the decision to prioritise time for data entry in PHIMS over other tasks. In such cases, the amount of time health promotion practitioners spent on administrative tasks significantly increased as a result of PHIMS implementation:

Darcey thinks it has been a bit of a shock to [the newly hired practitioner] to see how much of the job is data entry. (Fieldnotes, LHD D)

In other contexts, the division of labour changed in ways unanticipated by the designers of PHIMS. Sometimes, certain practitioners were delegated the responsibility for entering data into PHIMS for other practitioners. In one team, PHIMS data entry was mostly allocated to a single practitioner. Unlike other LHDs, practitioners in this team worked across services rather than being responsible for particular services for delivery of HCI programmes and so the way PHIMS is used has necessitated adaptation to align with their requirements. At another LHD, people spoke of how their team had always had a “PHIMS guru” who was an expert in the team. In teams such as these, a new range of skills, roles and responsibilities were subsequently required for some practitioners on an ongoing basis.

We found a key resource in the successful embedding of PHIMS was the provision of professional, effective and timely ongoing centralised technical support that is available to all PHIMS users, alongside ongoing investment in the continual development of the system. Many practitioners found the central technical support helpful and responsive for dealing with problems. PHIMS designers also recognised that creating new roles for PHIMS was essential when the technology was first introduced. Initial implementation across the state involved training one or two practitioners from each LHD in PHIMS as “PHIMS champions” who were responsible for training other team members.

At the time of fieldwork, many of the PHIMS champions had moved on and the role had not been replaced within the team. One of the original PHIMS champions explained the challenges of juggling the role with the “huge load” of programme delivery, describing how frequent staff turnover meant staff were upskilled as they came on board. In our observations of a PHIMS training with this PHIMS champion, she alternated between explaining the PHIMS system and her own personal approach to using PHIMS. Following the training, the team held a meeting in which all practitioners collaborated to develop a “minimum standard” for what they would enter into PHIMS. However, in other teams practitioners often described an ad-hoc approach to training:

"I came into the team and my orientation to PHIMS was, 'Here’s how you log on. It’s pretty self-explanatory. Just get in there and have a play.'That was pretty much my orientation to it.” (Interview, LHD G)

A perceived lack of sufficient ongoing training and exposure to PHIMS appeared to result in the inability of many practitioners to benefit from the system’s full functionality. Numerous practitioners expressed feelings of frustration, anxiety and mistrust in the system and felt that it contributed to a loss of productive work time:

She “is not too keen on PHIMS”, and she explains that the reason is that she uses the system irregularly and is not familiar with it, so she is worried that she messes up. (Fieldnotes, LHD M)

It is possible that a sense of inability to use or interact confidently with the technology, and uncertainty about the privacy/security of the data entered might have led to a default pattern of inaction by some people. Nevertheless, we observed that coordination and/or division of PHIMS labour arrangements enabled teams to comply with centrally mandated reporting requirements in PHIMS.

### Reflexive monitoring—appraising the value of PHIMS as an aid to practice

*Reflexive monitoring* refers to the opportunity an innovation provides the user to reflect on whether it improves/changes their practice. We used this construct to explore the extent to which embedding PHIMS enabled practitioners to articulate and compare “what is” with how health promotion “should be”. Some inbuilt aspects of PHIMS were obviously designed for this. For example, feeding back data from KPI achievement. Other reflexive monitoring opportunities happened by default, as any technical difficulties with PHIMS put the practitioner in the role of (instant) critic. In this sense, *reflexive monitoring* prompted us to consider how practitioners’ critiques of PHIMS and their suggestions for improving PHIMS intersected with the process of embedding. Notably, criticism of PHIMS did not equate to reflexivity. For reflexivity, frustration needed to be coupled with an articulation of what could be better and (hence) what is valued about practice.

Practitioners often came up with possible ways to re-design PHIMS and in doing so, conveyed what mattered to them in practice. For example, while PHIMS captured data based around individual schools and childcare services, some practitioners wanted to be able to capture and maintain records of contact with individual teachers they had built relationships with over time, particularly when those individuals changed jobs and moved between services. Other times, their practice needs were evident in the extra information management systems they devised to sit alongside PHIMS, as we describe elsewhere [20]. Practitioners also told us that PHIMS did not allow entry of information on all the groundwork, and therefore, underestimated, the work they did towards trying to achieve practice changes. This was problematic because PHIMS data could give a false impression of success as a site could appear to be going really well (according to the practice changes recorded) but actually be completely disengaged from the program. We describe this in a separate paper [22].

Some practitioners recognised benefits of PHIMS monitoring progress towards achieving health promotion goals in services that would otherwise take a long time to be visible:

"Especially health promotion which takes many years to show any sort of outcome … it [takes] a really long time to show any sort of improvement. So the good thing with PHIMS is that now we can show when we are getting better” (Interview, LHD D)

For others, however, it was clear that the implementation of PHIMS had altered the way health promotion was practised, compromising their interactions with schools:

Previously, the relationship was based on what the school wanted, now it’s based on what the health promotion officer needs for their job. (Fieldnotes, LHD I)

A newly graduated practitioner described how this approach to health promotion was markedly distinct from what she was taught at university:

They learned that each community is different and you have to take a very adaptable, tailored approach. However, with these programmes their hands are tied to delivering services in a very controlled way, where allowable adaptations are not meaningful. (Fieldnotes, LHD N)

Many practitioners expressed that the style of health promotion enshrined in PHIMS was a departure from that which was desired, and that practice was dominated by the imperative to collect data for PHIMS:

Stella says that their concern when PHIMS was launched was that the focus was going to be on achieving ticks in each box, on marking each practice off a list. This “horrified” her. They promised themselves they wouldn’t work this way. However, 3-4 years later, this is exactly how they work. (Fieldnotes, LHD I)

Engagement with the reporting parts of PHIMS was mandatory and part of service agreements between the LHDs and Ministry. For some teams the bare minimum of engagement with PHIMS—the work that *had to* be done for the Ministry—was enough. But they would not let it redefine their practice:

"I just feel that this is a mechanisation of things. I will do what it takes … Ok? If that makes everyone happy**.** But at the end of the day, it's not going to, and I won’t let it impact, on who I work with … We’ve got accountabilities that we need to stick to, because Ministry thinks that this is the way we should work, then we’ll do that." (Team Meeting, LHD B)

PHIMS directed practitioners to contact sites at specified time intervals (e.g. 6-monthly phone calls). One practitioner explained how time pressures impinged on her work by pushing to get information for deadlines:

"Sometimes it does feel like you’re just kind of surveying them and not actually doing health promotion." (Interview, LHD D)

Yet some practitioners embraced data collection as intrinsic to their role as a health professional:

"I’m hoping to [do more analysis of PHIMS data], because that would be a part of I guess, for myself as a health professional, to be able to get data and be able to analyse the data in a way that we can plan forward." (Interview, LHD H)

Some practitioners passionately denounced PHIMS/HCI as “not health promotion” or felt PHIMS was a “bureaucratic” add-on that got in the way of doing real health promotion work. In some LHDs, HCI practitioners were called “cookie cutters” by those working on other health promotion areas. This derogatory label implied that working on HCI was incongruous with health promotion principles and reflected a felt sense among many that health promotion encompassed autonomy, flexibility and creativity. In these ways, the criteria brought into the appraisal process are the definition of the profession and professional identity.

One practitioner with long experience in community development, described how she was able to strike a balance between the structured approach to HCI programme implementation underpinned by PHIMS, and meaningful practice:

Rose says that the bones [of the programs] are amazing. As a community developer in [other place] they did mainly the same things, but without the structure and the KPIs, it frankly made them untidy. So the current [structure] that the HCI programs offer is a huge value. So it is great, but Rose emphasizes that you get out what you put in. You can go there with a list and just check off the boxes and have a little chat and not make a difference. Or you can do what Rose feels like she is doing, go in, in depth and raise the issues from the core, with the monitoring guide as just a baseline … when Rose interviews, she counsels. (Fieldnotes, LHD L)

## Discussion

The ethnographers felt they gained in-depth access to most sites. However, a limitation of the “snapshot” time period meant that we were not able to observe first-hand all the activities that might allow us to appreciate embedding. Nor did we witness, what happened at the time of PHIMS’ first introduction. That said, participants often described in detail previous events, places and times, allowing us to construct the picture we give here.

NPT invited us to look at how PHIMS made sense and whether meaning was internalised, how PHIMS engaged users and how involvement was sustained, how PHIMS structured work individually and collectively and how PHIMS was appraised overall. We found a spectrum of PHIMS use. NPT allowed us to consider and label different ways in which PHIMS became integrated into practice. Other than simply “using” PHIMS, it helped us see roles that had been created, the practices that had been changed, the way time had become restructured, and how it crept into language/jokes. However, we cannot say that a non-NPT analysis of embedding (a grounded theory approach) would have been better (or worse) or different. Like MacFarlane and O’Reilly-de-Buin [36], we saw the economy of the structured approach, given that this was just one part of the analysis of a large data set with multiple concurrent analyses taking place. But we can say that the categories provided by NPT resonated with PHIMS designers and state-level managers, prompting a conversation about collective action being ongoing, consistent with the way NPT has embraced complexity [7]. That is, an effective system is not “set and forget” but in continuous evolution. People’s connection with it, and value from it, is dynamic.

The processes of embedding looked different within each LHD. All teams participated in PHIMS in some way, as collecting data about how compliant schools are with obesity prevention practices is mandatory to the local health team funding agreement. However, there were a variety of management approaches to incorporating PHIMS according to the attitude of the local HCI manager. Some managers encouraged practitioners to embrace the suggested schedule of activities for rolling out the programme and checking on sites and these managers relied heavily on the reporting functions in PHIMS. In some teams, the manager encouraged only minimal participation in PHIMS. In other teams there was participation but no overall cohesive team approach. And within many teams, individuals varied. For instance, in one LHD where the manager was firm of the view that PHIMS is “not what we do” (in terms of how they define health promotion), the team was directed to strategically work around PHIMS as a way of structuring practice. Indeed, in our original grounded theory codes (Appendix 1) workarounds happened often enough to be a sub-code. Yet it was also within this same team that one practitioner described how PHIMS was “quite useful” for recording notes and documenting her practice in one place.

So even where teams resisted relying on PHIMS as a way of informing their approach to practice, practitioners still used it as a documentation tool. Likewise, in another LHD, there was a strong emphasis on using PHIMS as a legitimate form of tracking progress. Indeed, a spirit of competition around achieving results in PHIMS had arisen, which was relished by some but was seen as less helpful by others [21]. So, while we observed that a team manager’s approach to PHIMS could encourage a certain kind of embedding, this may not support a practitioner’s individual practice needs. In the final quote of the findings, we see how an experienced practitioner—Rose—is able to fluidly organise and guide her practice by using PHIMS monitoring guide as “a baseline” and then go beyond this to tailor an in-depth and meaningful approach to her practice.

Technical difficulties or insufficient training with PHIMS risked disengagement and less use. This could, in theory at least, be an “easy fix” for ongoing training and update of PHIMS software design. This would increase engagement or the NPT dimension of cognitive participation. Non-participation in PHIMS by some practitioners is embedded by others doing the data entry for them. This is convenient and expedient, and as long as implementation targets are being reported as reached, few could argue it is a problem. Another reason why PHIMS endures may be that extra informal knowledge management systems have grown up around it [20]. Extra work is going into extra methods of recording. While some efficiencies could be made, the significance of this is that practitioners are propelled to do things well. The workarounds allow the technology to live and revealing a deep kind of professionalism [37].. Indeed, our overwhelming finding is that practitioners care about the way health promotion is done. Even where the “mechanisation” or over-standardisation of health promotion is seen as a threat, practitioners follow the Ministry’s request and manage to practise health promotion in ways that are most meaningful to them.

In terms of coherence, or the sense PHIMS makes to users, our study found that coherence might be improved if more users of the data understood how it is used at other levels of the system. This is illustrated best in the conversation “Don’t you think you would use PHIMS better if you knew that the Ministry was able to get more money for HCI because of it?” Again, this would seem at face value, an easy fix. Feedback of some of our preliminary findings to PHIMS designers and administrators also saw them identify better communication as a possible solution [26].

Yet these issues are complicated by the larger finding about PHIMS coherence to practice and the appraisal it receives (reflexivity). Practitioners and teams position themselves differently about PHIMS, and this plays out in a multitude of ways. It may affect their willingness to engage with training and feedback processes, it may colour their perception of communication from the Ministry, and it affects how they integrate PHIMS in their programme delivery approach. This hybrid mosaic is accepted and can possibly be seen as an enduring part of health promotion itself, i.e. debates on risk factor health promotion ‘versus’ community development [38]. Here one school of thought is that health promotion should focus on factors, like physical activity and nutrition, while the other sets out to address more primary determinants of health, such as social exclusion and collective empowerment. There are some practitioners who wistfully refer to the (more community development oriented) past and now confess that that they are now compliant with a system that once “horrified” them, while others welcomed the change. The level of fluid integration, as achieved by the experienced practitioner, Rose, may be more possible in smaller LHDS, because we found that in larger LHDs (with a large number of sites) PHIMS and HCI delivery adopted a divide-and-conquer imperative to get through the workload. It seems, therefore, that health promotion as a system in NSW has undergone a major transformation. The pattern is entrenched, and yet, it remains subject to all the push-and-pull tensions of top-down versus bottom-up health promotion. It remains subject to the ingrained checks and balances and self-criticisms that the field has had historically [39].

The pressure for diversity in health promotion practice therefore continues and can be considered a sign of resilience. It likely helped that a minimal level of engagement was possible in PHIMS, allowing local adaptation to occur. If scale-up was ever intended to be everyone doing the same thing in the same way, then PHIMS and the HCI would likely have failed. Indeed, the pattern of both adoption and non-adoption of technology within the context of programme scale-up was nuanced and complex. PHIMS’ endurance as part of a prevention “delivery” system appears to coincide with the phenomenon of self-organisation, which is consistent with complexity-informed understandings of scale-up in health care [40, 7, 41].

## Conclusion

Although the use of the software for data gathering and reporting was mandatory, the further adoption of PHIMS beyond this for guiding programme delivery was likely due to the flexibility allowed to individuals and teams and the local coordination and the respect accorded to different choices. The decades-old tension between top-down versus bottom-up styles of health promotion remains part of the new system of practice. Non-adoption was not simply failure to adopt, but a conscious position linked to primary professional values rooted in “bottom-up” practice. Others have argued that because self-organisation is inherent in complex systems, scale-up efforts that acknowledge self-organisation will be more likely to succeed than scale-up attempts that ignore it [40]. Thus, PHIMS’ endurance over 4 years may have been due to this hybrid mosaic pattern of self-organisation. Continued PHIMS use, and continued adjustments and renewal of personal and team-level choices of how to engage with it, could be facilitated by greater communication—not simply about PHIMS’ functionality or purposes (as conceived by its designers and state-level users) but to specifically encourage interaction and sensemaking among across user teams operating in diverse contexts, as the system of practice evolves.

## Data Availability

The dataset is not publicly available due to information that could compromise research participant consent and privacy.

## Appendix 1

Table 2

**Table 2:** “PHIMS” node and sub-nodes from the project codebook

	Name of node
1.	PHIMS
1.1	Approaches
1.1.1	Purpose
1.1.2	Roles and approach of PHIMS users
1.1.3	Team approaches
1.1.4	Training
1.2	Aspects of functionality
1.2.1	Data Entry
1.3	Feelings about PHIMS
1.3.1	Comparing monitoring systems
1.3.2	Confusion
1.3.3	Perceptions and feelings about use of PHIMS data
1.4	Intersection of PHIMS and practice
1.5	PHIMS use and data quality
1.6	Scheduled follow-ups
1.7	Tools and methods to organize practice
1.7.1	Workarounds

## Appendix 2

Table 3

**Table 3:** Normalisation Process Theory (NPT) coding template

NPT social mechanism	Description of construct within NPT social mechanism
**Coherence (sensemaking work)** Sensemaking work that is undertaken individually or collectively when attempting to incorporate a new set of practices into existing activities. Defines and organises the objects of a practice. (Do people know what the work is?)	**Differentiation:** How people come to an understanding about how sets of practices and their objects are different to each other.
	**Communal specification:** How people work together to build a shared understanding of the aims, objectives, and expected benefits of a set of practices.
	**Individual specification:** The work individuals do to help them understand their specific tasks and responsibilities around a set of practices.
	**Internalisation:** The work people do to understand the value, benefits and importance of a set of practices.
**Cognitive participation (commitment/engagement/buy in work)** The relational work people do to build and sustain commitment to a community of practice around a new set of practices - the enrolment and engagement of participants in a practice. (Are people prepared to join in with the work practice?)	**Legitimation**: Participants work to produce agreement about the legitimacy of a new set of practices. Do individuals believe it is right (legitimate) for them to be involved? Do they feel they can make a valid contribution to implementation?
	**Enrolment:** Taking account of whether or not key participants are working to initiate new practices.
	**Initiation:** Participants work to bring a new set of practices into being in specific times and places, investing commitment as they introduce it in practice.
	**Activation:** Participants work to produce and reproduce continued commitment to new practices, sustaining involvement in new practices. PHIMs in health promotion practice, sustaining involvement in monitoring of health promotion practice over time.
**Collective action (enacting work)** Purposive, operational work that defines and organises the enacting of a practice - interaction with already existing practices; Knowledge work that people do to build accountability and maintain confidence in a set of practices and in each other as they use them. (How do people do the work?)	**Contextual integration**: The capacity of an organisation to understand and agree to the allocation of resources, infrastructure and policy in order to implement a complex intervention, and to negotiate its integration into existing patterns of activity. Proposition: Normalisation is likely if it confers an advantage on an organisation in flexibly executing and realising work.
	**Relational integration** ^a^ **:** (the embeddedness of trust in professional knowledge and practice). The network of relations in which encounters between agents (professionals and clients) are located, and through which knowledge and practice relating to a complex intervention is defined and mediated. Proposition: Normalisation is likely if it equals or improves accountability and confidence within networks.
	**Interactional workability:** The interaction of agents (professionals, clients, others) in operationalising a complex intervention. Proposition: Normalisation is likely if it confers an interactional advantage in flexibly accomplishing congruence (co-operation and legitimacy) and disposal (shared expectations about goals, meaning and outcomes of the new practices).
	**Skill set workability**: The organisational distribution of work, knowledge and practice across divisions of labour. The formal and informal divisions of labour in health care settings, and the mechanisms by which knowledge and practice about complex interventions are distributed. Proposition: Normalisation is likely if a complex intervention is calibrated to an agreed skill-set at a recognisable location in the division of labour.
**Reflexive monitoring (appraisal work)** The appraisal work that people do to assess and understand the ways that a new set of practices affect them and others around them, including determining how effective and useful the new practices are for themselves and others. Information is gathered both experientially and systematically by individuals and by formal and informal groups to evaluate practices. This work may lead to attempts to redefine procedures or modify practices or the new technology itself.	**Systematisation:** Participants work to define, collect, and collate information about the effects of new practices.
	**Communal appraisal:** Participants work to assess the collective utility and value of new practices according to their place in the healthcare system.
	**Individual appraisal:** Participants work to appraise their experience of the value of new practices.
	**Reconfiguration**: Participants work to inform changes in patterns of participation and action, informing changes in the way that new practices are enacted.

^a^Interactional workability and relational integration: These constructs referred to the professional-patient interaction, and the degree to which normalisation of an intervention could occur was dependent on whether this interaction was disrupted or whether confidence in the knowledge and practice that underpinned it was undermined.

## Abbreviations

PHIMS: Population health information monitoring system
HCI: Healthy Children’s Initiative
LHD: Local health district
NSW: New South Wales
